# A Protocol for Transverse Cardiac Slicing and Optical Mapping in Murine Heart

**DOI:** 10.3389/fphys.2019.00755

**Published:** 2019-06-25

**Authors:** S. He, Q. Wen, C. O’Shea, R. Mu-u-min, K. Kou, A. Grassam-Rowe, Y. Liu, Z. Fan, X. Tan, X. Ou, P. Camelliti, D. Pavlovic, M. Lei

**Affiliations:** ^1^Key Laboratory of Medical Electrophysiology of Ministry of Education and Medical Electrophysiological Key Laboratory of Sichuan Province, Institute of Cardiovascular Research, Southwest Medical University, Luzhou, China; ^2^Institute of Cardiology, Union Hospital, Tongji Medical College, Huazhong University of Science and Technology, Wuhan, China; ^3^Institute of Cardiovascular Sciences, University of Birmingham, Birmingham, United Kingdom; ^4^Department of Pharmacology, University of Oxford, Oxford, United Kingdom; ^5^Department of Cardiovascular Medicine, Southwest Medical University, Luzhou, China; ^6^School of Biosciences and Medicine, University of Surrey, Guildford, United Kingdom

**Keywords:** optical mapping, transverse cardiac slice, membrane potentials, Ca^2+^ transients, murine heart

## Abstract

Thin living tissue slices have recently emerged as a new tissue model for cardiac electrophysiological research. Slices can be produced from human cardiac tissue, in addition to small and large mammalian hearts, representing a powerful *in vitro* model system for preclinical and translational heart research. In the present protocol, we describe a detailed mouse heart transverse slicing and optical imaging methodology. The use of this technology for high-throughput optical imaging allows study of electrophysiology of murine hearts in an organotypic pseudo two-dimensional model. The slices are cut at right angles to the long axis of the heart, permitting robust interrogation of transmembrane potential (V_m_) and calcium transients (CaT) throughout the entire heart with exceptional regional precision. This approach enables the use of a series of slices prepared from the ventricles to measure V_m_ and CaT with high temporal and spatial resolution, allowing (i) comparison of successive slices which form a stack representing the original geometry of the heart; (ii) profiling of transmural and regional gradients in V_m_ and CaT in the ventricle; (iii) characterization of transmural and regional profiles of action potential and CaT alternans under stress (e.g., high frequency pacing or β-adrenergic stimulation) or pathological conditions (e.g., hypertrophy). Thus, the protocol described here provides a powerful platform for innovative research on electrical and calcium handling heterogeneity within the heart. It can be also combined with optogenetic technology to carry out optical stimulation; aiding studies of cellular V_m_ and CaT in a cell type specific manner.

## Introduction

Thin living tissue slices are a widely used experimental preparation for electrophysiological studies of the brain (Accardi et al., 2016). Slices have recently been adopted as a promising model for the investigations of cardiac electrophysiology in different species, for example: viral delivery, culturing, and mechanical stimulation (Halbach et al., 2006; Bussek et al., 2009; Camelliti et al., 2011; Wang et al., 2015; Kang et al., 2016; Thomas et al., 2016; Randi et al., 2017; Watson et al., 2017, 2019; Wen et al., 2018). Importantly, cardiac slices exhibit similar electrophysiological characteristics to the intact heart and respond to the application of pharmacological compounds similarly to the whole heart (Bussek et al., 2009; Camelliti et al., 2011; Wen et al., 2018). They have advantages for investigating transmural and regional characteristics of a variety of physiological parameters of the heart, thus providing a promising experimental model for heart research.

Because of feasibility for genetic modification, the mouse has been widely used for exploring molecular, cellular and systemic mechanisms underlying inherited and acquired cardiac arrhythmic diseases (Nerbonne and Kass, 2005; Huang, 2017). Despite the popularity of mouse models in cardiac arrhythmic research, cardiac transmural heterogeneities of action potential and Ca^2+^ transient characteristics, which are vitally important for phenotypic and mechanistic research in cardiac disease conditions, have only been explored recently in the mouse heart (Wen et al., 2018). We reported a robust approach for transverse cardiac slicing and optical mapping of transmembrane potential (V_m_) and intracellular Ca^2+^ transient (CaT) in murine hearts, providing unprecedented and potentially high-throughput characterization of action potentials (APs) and intracellular Ca^2+^ transients *everywhere* in the mouse ventricles (Wen et al., 2018). Such an approach can be potentially adapted for large mammalian hearts and human tissue, thus representing a powerful *in vitro* model system for translational cardiovascular research (Thomas et al., 2016; Randi et al., 2017; Watson et al., 2017, 2019). Using this technique, we demonstrated the feasibility of our new approach to cardiac slicing for systematic analysis of intrinsic transmural and regional gradients in V_m_ and CaT in murine ventricular tissue (Wen et al., 2018).

In the present protocol, we describe a detailed feasible transverse slicing method, cutting slices at right angles to the long axis of the heart and combined it with a high-throughput optical imaging technique as a new approach for studying cellular electrophysiology of murine heart in an organotypic pseudo two-dimensional ventricular tissue model. Our method enables the use of a series of slices prepared from the ventricles to simultaneously measure V_m_ and CaT with high temporal and spatial resolution allowing comparison of transmural and regional profiles of APs and CaTs. This technique can potentially be combined with other molecular techniques such as *in situ* immunostaining and virus transfection to gain insight into arrhythmia mechanisms in various heart disease conditions.

## Materials and Equipment

### Animals

Animals used were 10–12 weeks, 20–25 g, male C57BL mice or Pnmt-Cre/ Channelrhodopsin 2 (ChR2) mice as we previously reported (Wang et al., 2017). The Pnmt-Cre/ChR2 mouse line exhibits cell-type specific expression of ChR2 by crossing B6.Cg-*Gt (ROSA)26Sor^tm27.1(CAG–COP4H134R/tdTomato) Hze^*/J strain (Stock No. 012567, Jackson Labs) with a Cre transgenic strain under the control of a *Pnmt* promoter (Wang et al., 2017). ChR2 was specifically introduced into murine cells expressing the *Phenylethanolamine n-methyltransferase* (*Pnmt*) gene, which encodes for the enzyme responsible for conversion of noradrenaline to adrenaline. The murine model led to the identification of a distinctive class of Pnmt-expressing neuroendocrine cells and their descendants (i.e., Pnmt^+^ cell derived cardiomyocytes) within the heart (Wang et al., 2017).

All procedures including animal subjects have been approved by Institutional Animals Ethics Committees at Southwest Medical University, Luzhou, China or Department of Pharmacology at University of Oxford, United Kingdom and the national guidelines under which the institution operates. All mice used in this study were maintained in a pathogen−free facility at Southwest Medical University or University of Oxford. Mice were given *ad libitum* access to food and water. The authors confirm that they have taken all steps to minimize the animals’ pain and suffering.

### Chemicals and Reagents

The relevant information of the chemicals and reagents are described in Table 1.

**TABLE 1:** Reagents utilized.

**Chemical and catalog references**	**Supplier**
NaCl (SLBS2340V)	Sigma-Aldrich, St. Louis, MO, United States
NaHCO_3_ (SLBX3605)	Sigma-Aldrich
NaH_2_PO_4_ (BCBW9042)	Sigma-Aldrich
KCl (SLBS5003)	Sigma-Aldrich
MgCl_2_ (BCBS6841V)	Sigma-Aldrich
CaCl_2_ (SLBK1794V)	Sigma-Aldrich
Glucose (SLBT4811V)	Sigma-Aldrich
HEPES (W1122DO10)	Sangon biological, Shanghai, China
2,3-butanedione monoxime (BDM) (29297)	Sigma-Aldrich
Blebbistatin (SLBV5564)	Tocris Bioscience, Minneapolis, MN, United States
Voltage-sensitive dye RH237 (1971387)	Thermo Fisher Scientific, Waltham, MA, United States
Calcium indicator Rhod-2AM (1890519)	Invitrogen, Carlsbad, CA, United States
Dimethyl sulfoxide (DMSO) (RNBT7442)	Sigma-Aldrich
Heparin Sodium (H51021209)	Chengdu Haitong Pharmaceutical Co., Ltd., Chengdu, China
Avertin (2,2,2-tribromoethanol)	Sigma-Aldrich Poole, Dorset, United Kingdom
Pluronic F127 (1899021)	Invitrogen, Carlsbad, CA, United States
Low-melt agarose (A600015.0005)	BBI Life Sciences, Shanghai, China

### Instruments and Equipment

- Dumont forceps (Medical Equipment Factory of Shanghai Medical Instruments Co., Ltd., Shanghai, China).
- Mayo scissors (Medical Equipment Factory of Shanghai Medical Instruments Co., Ltd., Shanghai, China).
- Kelly hemostatic forceps (Medical Equipment Factory of Shanghai Medical Instruments Co., Ltd., Shanghai, China).
- Iris forceps (Medical Equipment Factory of Shanghai Medical Instruments Co., Ltd., Shanghai, China).
- Syringe Filter, Aquo-system, 0.45 μm/13 mm (Sangon Biotech Shanghai, China, F513143-0001).
- Langendorff perfusion system.
- Perfusion pump (Longer Precision Pump Co., Ltd., Baoding, China, BT100-2J).
- Vibration slicer (Leica VT1000s, Nussloch, Germany).
- EMCCD camera (Evolve 512, Photometrics, Tucson, AZ, United States).
- MacroLED light source (Cairn Research, Faversham, United Kingdom).
- MyoPacer EP field stimulator (Ion Optix Co., Milton, MA, United States S006152).
- Electrical stimulation chamber (custom-made by the workshop of Department of Pharmacology, University of Oxford).
- Heater Controller (custom-made by the workshop of Department of Pharmacology, University of Oxford).

### Solutions

- Krebs solution (containing in mM: NaCl 119, NaHCO_3_ 25, NaH_2_PO_4_ 1.0, KCl 4.7, MgCl_2_ 1.05, CaCl_2_ 1.35, and glucose 10; equilibrated with 95% O_2_/5% CO_2_, pH 7.4) at 37°C, pH = 7.35–7.4.
- HEPES buffered BDM solution (in mM: NaCl 140; KCl 4.7; glucose 10; HEPES 10; MgCl_2_ 1.05; CaCl_2_ 1.35; pH 7.4) containing the excitation–contraction uncoupler 2,3-butanedione monoxime (BDM, 10 mM) and was stored at 4°C.
- Blebbistatin stock solution, 10 mM in DMSO.
- Voltage-sensitive dye RH237 stock solution, 1.25 mg/mL in DMSO.
- Calcium indicator Rhod-2AM stock solution, 1 mg/mL in DMSO.

### Preparation of Experimental Setup(s)

**FIGURE 1:**
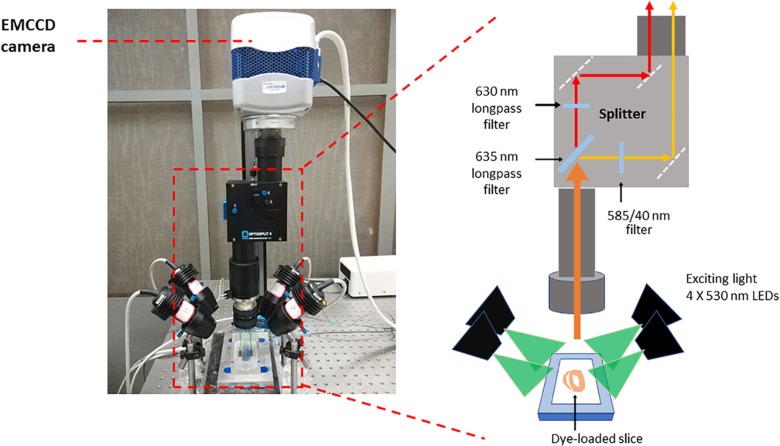
Optical mapping set-up. The system consists of a camera (Photometrics Evolve Delta 512) running under Metamorph (Molecular Devices) in Light-speed mode, giving a high temporal resolution of sub-frames, up to 1000 frames/sec, and spatial resolution at the sample of 74 × 74 μm per pixel. The sample was imaged using a custom MacroScope (Cairn Research) with an F/0.95, 25 mm C-mount camera lens, spaced so as to give a working distance of approximately 40 mm. The excitation light was provided by a four light emitting diode MacroLED lamps (525 nm, 1750 lumen, 7 mm^2^ emitters, Cairn Research) with aspheric condensers directed onto the slices from the four corners of the bath, so as to produce an even, near-critical, illumination at approximately 30 mm. The LEDs were driven using MacroLED control boxes and digitally modulated from Metamorph software with a National Instruments multifunction card. The LEDs were individually filtered using ET525/50 sputter coated filters (Chroma Technology) to remove any out-of-band light. The fluorescence emission light was split with a 610 nm long-pass dichroic mirror and corresponding emitters to separate the Ca^2+^ and voltage signals based on their wavelengths. The voltage-sensitive dye, RH237, emits signals which exhibit a peak at 670 nm, while Rhod-2 AM has a peak emission at approximately 600 nm and, once split by the dichroic mirror, these two signals are imaged on to the camera such that they are reproduced side-by-side on the sensor and recorded simultaneously. This dichroic and adjustable mirror unit, the OptoSplit, was provided by Cairn Research with filters from Chroma Technologies (United States). CaT fluorescence was collected at 585 ± 40 nm and Vm using a 662 nm long pass filter. Vm and CaT measurements were taken at maximal resolution (128 × 128 pixels; pixel area 74 × 74 μm) at a rate of 1000 frames/s.

- Prepare the Langendorff perfusion system. Turn on the perfusion system pump. Starting the peristaltic pump that is used for retrograde perfusion. Wash the perfusion system with 1 L deionized water with initial flow rate at 90 ml/min. Once all the deionized water has evacuated from the chamber, circulate the Krebs solution and pass it through a 0.45 μm filter. Keep Krebs solution at 37°C with a water jacket, circulated and oxygenated by bubbling O_2_/CO_2_ (95%/5%) gas into the solution. Adjust the flow rate to 4 ml/min.
- Prepare the optical mapping system (Figure 1), a custom-designed system equipped with an EMCCD camera. The excitation light for Ca^2+^ sensitive dye Rhod-2 and voltage sensitive dye RH237 is provided by four MacroLED lamps (525 nm, 1750 lumen, 7 mm^2^ emitters, Cairn Research) with aspheric condensers driven using MacroLED control boxes and digitally modulated from Metamorph software. LEDs are directed onto the slices from the four corners of the bath, to produce an even, near-critical, illumination at approximately 30 mm, with equal distance from 4 corners. An ET525/50 sputter coated filter (Chroma Technology) is used to remove any out-of-band light for each LED.
  Samples are imaged using a custom MacroScope (Cairn Research) with an F/0.95, 25 mm C-mount camera lens, spaced to give a working distance of approximately 40 mm. The fluorescence emission light is split with a 635 nm longpass dichroic mirror and subsequently filtered (Vm: 662 nm LP, CaT: 585/40 nm) to separate the Ca^2+^ and voltage signals based on their wavelengths using the OptoSplit apparatus (Cairn Research). Once split, the Vm and CaT signals are imaged on to the camera such that they are reproduced side-by-side on the sensor and recorded simultaneously (Vm imaged on one side of the chip, CaT on the other).
- Prepare the vibratome. Install a new blade at the holder and fill the cooling chamber with crushed ice.

## Procedure

**FIGURE 2:**
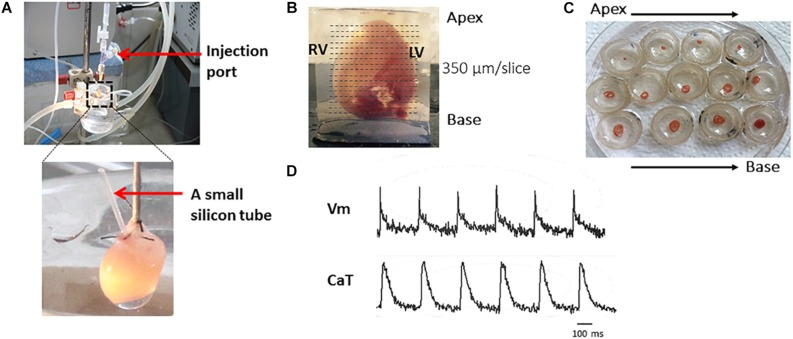
The major steps of the protocol. Representative harvested mouse heart and transverse slices from apex to base. Briefly, after the Langendoff perfusion steps **(A)**, embed the heart in 4% low-melting agarose and cut with right angle to the long axis by Leica VT1000s vibratome **(B)**. Slices are transferred to petri dishes **(C)** and voltage and calcium transients recorded **(D)**.

Figure 2 shows the major steps of the protocol.

### Harvest, Cannulation, and Perfusion of Mouse Heart (30 min)

(1) Anesthetize the animal with 1.2% Avertin solution (0.5–0.8 ml I.P.) containing heparin (200 units I.P.), ensuring lack of pain reflex, prior to sacrifice (Complete in 10 min).
(2) Open the chest, quickly remove the heart and keep the heart in oxygenated (95% O_2_, 5% CO_2_), (37 ± 1°C) Krebs solution. Using a dissecting microscope, identify the aorta and make a clean cut across the ascending aorta below the right subclavian artery. Cannulate the aorta onto a custom-made cannula and tie with 4-0 black-braided silk suture around the cannula. After cannulation, retrogradely perfuse the heart and superfuse with constant-temperature (37 ± 1°C) Krebs solution. The retrograde perfusion rate was adjusted in the range of 3.5–4.0 mL/min (Complete this step in 3 min).
(3) Important! Insert a small silicon tube (0.7 mm diameter) into the left ventricular cavity through the left atrial appendage in order to prevent ventricular pressure increasing due to solution congestion and to prevent acidification of the perfusate trapped in the left ventricular cavity. This is especially important after the suppression of ventricular contractions with an excitation-contraction uncoupler.

After the dyes are loaded, the steps described in Sections “Chemicals and Reagents,” “Instruments and equipment,” and “Solutions” should be performed in the dark.

### Load Calcium and Voltage Sensitive Dyes and Excitation-Contraction Uncoupler (50–60 min)

(1) Before dye loading, ensure the isolated heart is beating rhythmically without ischemia (10 min).
(2) Mix 50 μl calcium indicator Rhod-2 AM 1:1 with Pluronic F127 (20% solution in DMSO) and then dilute in 1 ml Krebs solution. Recirculate oxygenated (95% O_2_, 5% CO_2_) Krebs solution at constant-temperature (37 ± 1°C) and then inject Rhod-2 AM solution slowly over 7–10 min through the injection port near the cannula.
(3) Continue to recirculate Krebs solution after finishing injection (now, the perfusate contains Rhod-2 AM and Pluronic F127) (20 min).
(4) During this time, dilute 30 μl voltage-sensitive dye RH237 (1.25 mg/ml stock) in 1 ml Krebs solution.
(5) Switch to perfusion with oxygenated (95% O_2_, 5% CO_2_), constant-temperature (37 ± 1°C), Krebs solution and perfuse the heart for 2–3 min. Then inject RH237 solution slowly over 7–10 min through the injection port.
(6) After loading the voltage dye, recirculate oxygenated Krebs solution containing 10 μM blebbistatin [maintaining constant temperature at 37 ± 1°C] until the heart stops beating (10–15 min).
(7) Whilst waiting for the heart to stop beating, prepare equipment and solutions for ventricular slicer. A standard razor blade is cut into half and placed on the blade holder so that the blunt side of the blade was tight up against the holder. A circle of solidified agar is mounted on the ventricular slicer’s plunger using superglue.

### Ventricular Slicing, Harvest, and Recovery (60–70 min)

(1) Take the heart off the Langendorff setup.
(2) Carry out all dissections in ice cold HEPES buffered BDM solution. Remove atria and connective tissue from the ventricles to flatten the base.
(3) Mount base of the heart on solidified 4% agarose of slicer’s plunger using surgical glue.
(4) Freshly prepare 4% low-melt agarose, cool on ice, and pour to cover the whole heart. Surround the plastic cap with the metal support.
(5) After the agarose is solidified, place the ventricular slicer plunger inside the metal merged into vibratome bath until it reaches stop point. Fill the ventricular slicer bath with ice-cold HEPES buffered BDM solution.
(6) Cut slices transversally at a thickness of 300–350 μm, with speed of 0.05 mm/s, amplitude of 1 mm and vibration frequency of 80 Hz (2 min per slice).
(7) On average 14 ventricular slices are harvested. Transfer each slice (other than the first which was usually discarded) to a petri dish containing ice-cold HEPES buffered BDM solution for 10 min. Pin the slices on small pieces of thin square shape Sylgard sheets (1 × 1 cm in diameter, 0.2 cm thickness), cover with mesh. Then transfer the slices to 1st, 2nd, and 3rd petri dishes filled with oxygenated blebbistatin Krebs solution (10 μM) at room temperature (RT) for 10 min in each dish.
(8) Repeat slicing procedure until the last slice is harvested.

### Optical Mapping

(1) Place each slice in oxygenated blebbistatin Krebs solution (10 μM) in 37°C tissue bath for 2–3 min to equilibrate.
(2) Place the slice into a custom-made stimulation chamber filled with 37°C oxygenated blebbistatin Krebs solution (10 μM) between the two pacing electrodes. Use pin(s) and mesh to flatten the slice.
(3) Place the slices into the optical mapping setup (Figure 2, details in Materials and equipment). For field pacing, stimulate with square pulses of 2 ms duration, at voltages 1.5 times above threshold (minimum voltage required to pace tissue) with 1:1 coupling.
(4) For optical pacing, the tissues are paced through the activation of ChR2 light-sensitive channels. This is achieved by the delivery of 470 nm blue light pulses (2–10 ms pulse width) generated by OptoFlash (Cairn Research, Faversham, United Kingdom). Pulses are triggered by a 1401 digitiser and Spike 2 software (Cambridge Electronic Design). Approximate blue light intensity is measured with a 818-ST2 Wand Detector connected to a 843 R Power meter (both Newport Corporation, CA, United States) and we estimate an average irradiance in our experiments of 0.1–0.3 mW/mm^2^ based on an approximate distance of 1–2 cm between Sylgard and liquid light guide (Oriel instruments Model No 77525).
(5) For AP and CaT alternans investigations, stimulate slices at frequencies of 2, 4, 8 and 16 Hz. Between pacing protocols, turn off the LED lights to minimize photobleaching (and other possible side effects, including phototoxicity). When testing, turn off the oxygenation to avoid motion artifacts.
(6) After experiment is finished, clean the perfusion system with deionized water.

### Data Analysis

(1) Recorded image files are loaded into an optical mapping analysis software ElectroMap^1^ (O’Shea et al., 2019). Pre-process images with application of 3 × 3 pixel Gaussian spatial filter and top-hat (kernel length = 100 ms) baseline correction.
(2) Measure action potential duration (APD)/Calcium transient duration (CaTD) at desired repolarization/decay percentage at each pixel across the tissue, as measured from time of maximum upstroke velocity. Voltage-calcium latency can be measured as time difference between AP and CaT peak.
(3) For assessment of conduction velocity, render activation map by measuring activation time, for example by measuring depolarization midpoint or upstroke time (dV/dt max). Conduction velocity across the slice can then be quantified using multi-vector polynomial method with a local window size of 5 × 5 pixels (Bayly et al., 1998).

### Exemplar Results

Figure 1 shows the optical mapping set-up. After the slices were prepared, slices were pre-incubated at room temperature in Krebs solution containing 10 μM blebbistatin for an optimal recovery time, prior to electrophysiological assessment using a custom-designed optical mapping system.

Figure 2 shows the major steps of the protocol. To achieve optimal dye loading, the voltage dye RH237 and/or Ca^2+^ dye Rhod-2 AM were loaded through the coronary circulation using the Langendorff perfusion system (Figure 2A). Prevention of blood clot formation was essential for optimal dye loading and was achieved by initial injection of heparin (200**–**300 units) before animal sacrifice. Ventricles were embedded in low-melting temperature agarose as illustrated in Figure 2B to provide structural support to the tissue during sectioning. Slices were cut at right angles to the long axis of the heart from the apex to the base, and thicknesses from 300**–**350 μm found to be the best thickness to achieve optimal recordings for both Vm and CaT (Figure 2B). Figure 2C shows viable slices obtained with the optimized protocol and representative V_m_ and CaT signals.

**FIGURE 3:**
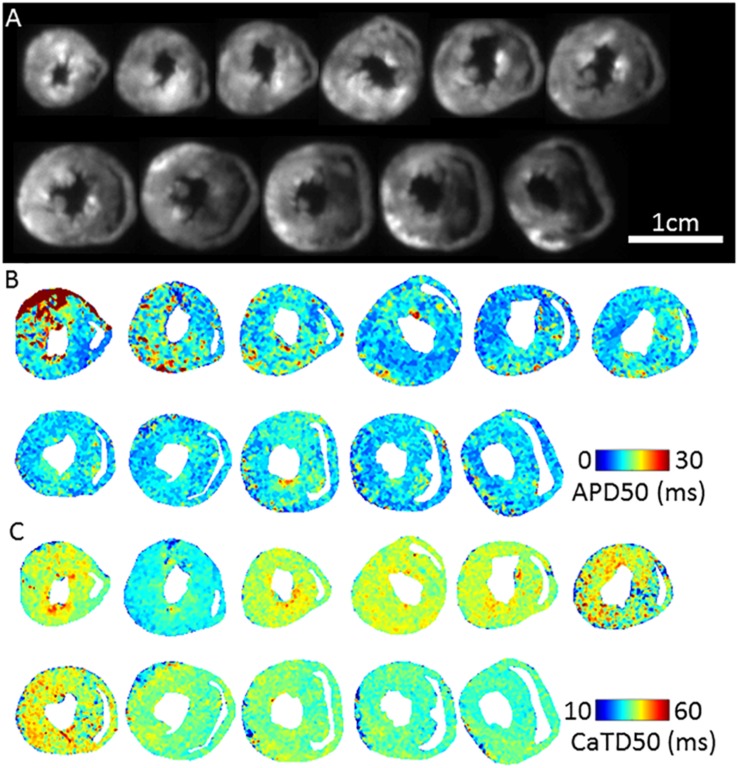
Dual Vm and CaT imaging in murine transverse ventricular slices. **(A)** Fluorescence image (voltage-RH237) of transverse slices of murine ventricles from apex to base, dual loaded with both transmembrane voltage (RH237) and intracellular calcium dyes (Rhod-2 AM). **(B)** Representative maps of AP duration (APD50) at 2 Hz pacing frequency (500 ms pacing cycle length) recorded from apex to base ventricular slices. **(C)** Representative maps of Calcium transient duration (CaTD50) at 2 Hz pacing frequency recorded from apex to base ventricular slices.

Figure 3A shows fluorescence images of voltage dye RH237 and Ca^2+^ dye Rhod-2 AM as imaged using the optical mapping setup. Using this protocol, and the optical imaging setup, we are able to dually measure parameters such as AP duration (Figure 3B) and CaT duration and (Figure 3C). The “rundown” of the Vm and CaT signals was monitored for up to 4 h, the average signal “rundown” being less than 25%. Our experiments usually finished within 3 h from the beginning of Langendorff perfusion of the heart.

**FIGURE 4:**
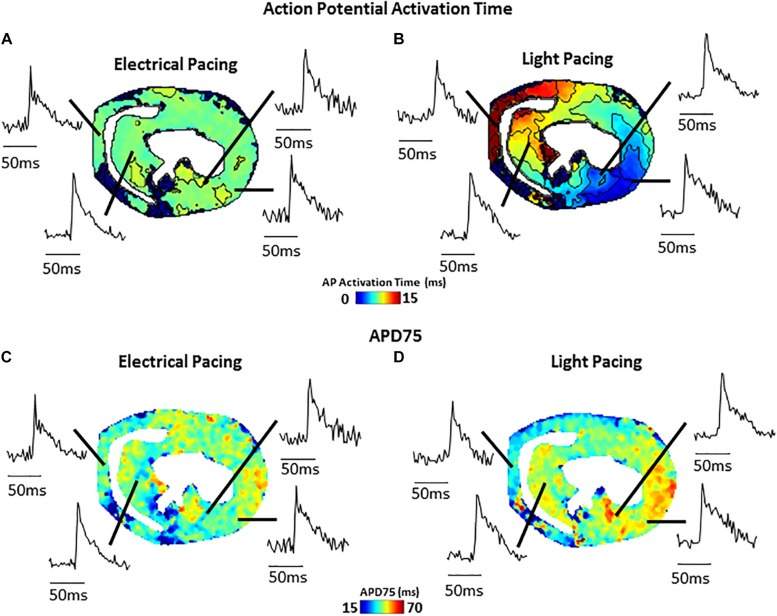
Comparison of electrical (field stimulation) and light (470 nm pulses) pacing in murine transverse ventricular slices. **(A)** Activation map and example signals (transmembrane voltage, RH237) from slice paced using electrical field stimulation. **(B)** Activation map and example signals from the same slice paced using blue light stimulation of ChR2. **(C)** APD75 map from slice during electrical field stimulation. **(D)** APD75 map from slice during blue light stimulation.

Figure 4 shows an example of activation (Figure 4A) and APD75 (Figure 4B) maps reconstructed from electrical and light-paced APs in a ventricular slice from Pnmt-Cre/ChR2 mouse heart. Also shown are exemplar V_m_ signals from different regions of the ventricle.

## Discussion

The protocol described here allows high resolution mapping of V_m_ and CaT on thin transverse slices cut at right angles to the long axis of the heart, permitting robust interrogation of V_m_ and CaT throughout the entire heart with exceptional regional precision. Since the apico-basal location of each slice is known and every slice is fully transmural, such rich data allows for characterization of regional AP and CaT properties. This includes AP and CaT durations, alternans threshold, transmural activation and conduction velocities, and correlation of AP and CaT properties such as voltage-calcium coupling latency in each slice. The main processes of the protocol include: (i) Preparing solutions and experimental setup; (ii) Harvesting, cannulation and perfusion of the mouse heart by Langendorff perfusion; (iii) Loading calcium and voltage sensitive dyes and excitation-contraction uncoupler; (iv) Ventricular Slicing; (v) Optical mapping; (vi) Image processing and analysis. The protocol was further developed since it was first published (Wen et al., 2018) in two major aspects: (i) simultaneous dual dye images allowing correlation of AP and CaT properties in each slice; (ii) implementing improved image processing and analysis such as correlation of AP and CaT properties.

### Applications

Our novel approach potentially provides not only a unique technique applicable to hearts of other species (such as widely used rat and rabbit), but also other tissues such as brain, adrenal medullary tissue, gut, etc. As we demonstrate here (Figure 4), such slice imaging technique can be also combined with optogenetic technology to carry out optogenetic light stimulation of specific cells. Such an approach could be further extended by use of genetically encoded reporter proteins allowing cell-type specific study of V_m_ and Ca^2+^ signals, as well as cell-specific activation. We believe that the approach would be of considerable interest to researchers in manifold biomedical research fields and would serve as a useful platform for further innovative biomedical research.

### Advantages

Firstly, a critical advantage of our cardiac slice model is the ability to provide access to any region of the ventricles, thus enabling exploration of regional and transmural differences. This is particularly important for the mouse heart, given that other *in vitro* tissue preparations, such as ventricular wedges, are not feasible for small hearts. Secondly, this technique may prove extremely valuable for characterizing regional arrhythmogenic changes in genetic murine models of cardiovascular disease (e.g., catecholaminergic polymorphic ventricular tachycardia, hypertrophic cardiomyopathy), as well as comprehensive regional characterization of remodeling in acquired pathologies (myocardial infarction, pressure-overload heart failure). Thirdly, depending on the optics, camera resolution, and slice size and position, it may be possible to image several or all slices from a single mouse heart simultaneously, giving a high throughput platform to electrophysiological investigations. Finally, it can also be combined with optogenetic technology to carry out optogenetic light stimulation – aiding studies requiring precise manipulation of cellular V_m_ and CaT.

### Limitations and Adaptations

(i) Possibility of tissue injury increasing regional Vm and CaT differences. This can be overcome by optimized protocol with very slow cutting speed, embedding in supportive but oxygen permeable medium, and use of blebbistatin as uncoupler during slice preparation. (ii) Rundown of the V_m_ and CaT signals. The “rundown” of the V_m_ and CaT signals was also monitored for up to 4 h, the average signal “rundown” being less than 25%. Our experiments usually finished within 3 h. This can be overcome by efficient imaging processes, and the application of 0.5 mM Probenecid in the recovery and recording solutions to reduce the Ca^2+^ dye loss. (iii) The measurements of the parameters under electrical pacing condition was conducted by field stimulation (considering the fragile dedicated slice preparation), thus our approach may suffer from tissue activation occurring at the same time point across the preparation. Whilst this is not what we observed regularly in our data, this approach may affect the conduction velocity (CV) measurement. A better approach with point stimulation or selective optical activation of a subset of cells (Figure 4) should be pursued.

## Ethics Statement

Animals used were 10–12 weeks, 20–25 g, male B57BL mice. All procedures including animal subjects have been approved by the Institutional Animals Ethics Committees at the Southwest Medical University, Luzhou, China or the Department of Pharmacology at the University of Oxford and the national guidelines under which the institution operates. All mice used in this study were maintained in a pathogen−free facility at the Southwest Medical University or the University of Oxford. Mice were given *ad libitum* access to food and water. The authors confirm that they have taken all steps to minimize the animals’ pain and suffering.

## Author Contributions

SH, QW, RM, XT, and XO carried out the experiments. CO’S and DP carried out the data process and data analysis. PC and ML designed the experiments. SH, QW, RM, XT, XO, and ML drafted the manuscript. PC, CO’S, and DP revised and edited the manuscript. All authors have made a substantial contribution to the manuscript.

## Conflict of Interest Statement

The authors declare that the research was conducted in the absence of any commercial or financial relationships that could be construed as a potential conflict of interest.

## Footnotes

1 https://github.com/CXO531/ElectroMap
